# Supplementary material for: A Novel Method for Achieving Precision and Reproducibility in a 1.8 GHz Radiofrequency Exposure System That Modulates Intracellular ROS as a Function of Signal Amplitude in Human Cell Cultures
Source: Bioengineering (Basel). 2025 Mar 4;12(3):257. doi: 10.3390/bioengineering12030257 (PMC11939444; doi:10.3390/bioengineering12030257)
Supplement: Supplementary file 1 [file bioengineering-12-00257-s001.zip › bioengineering-3458348-supplementary.pdf]

SUPPLEMENTARY INFORMATION.

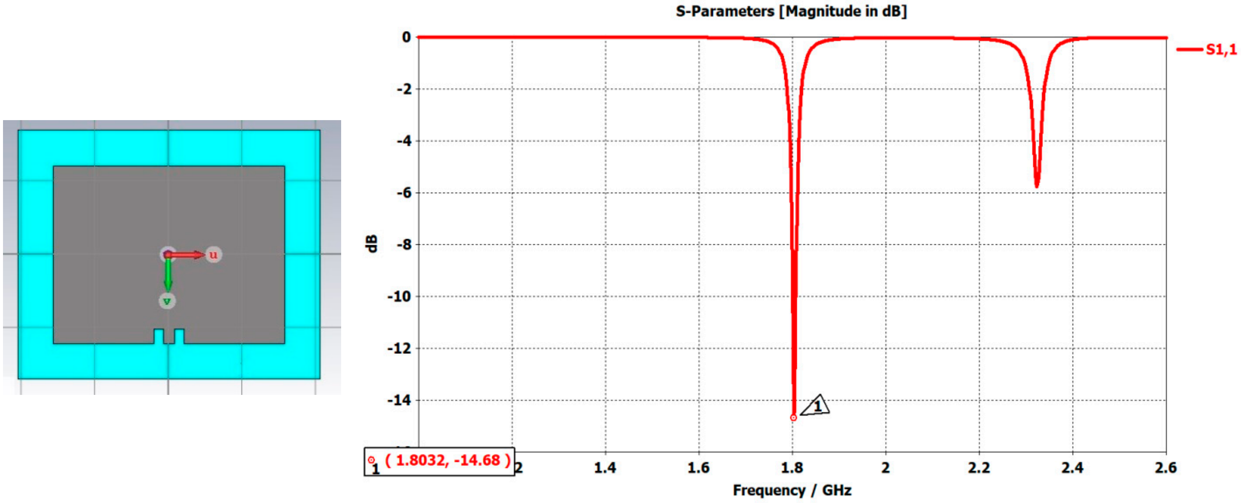

Figure S1: CST patch antenna design (left) and CST-stimulated reflection coefficient (right), S11=-14.68@1.80GHz

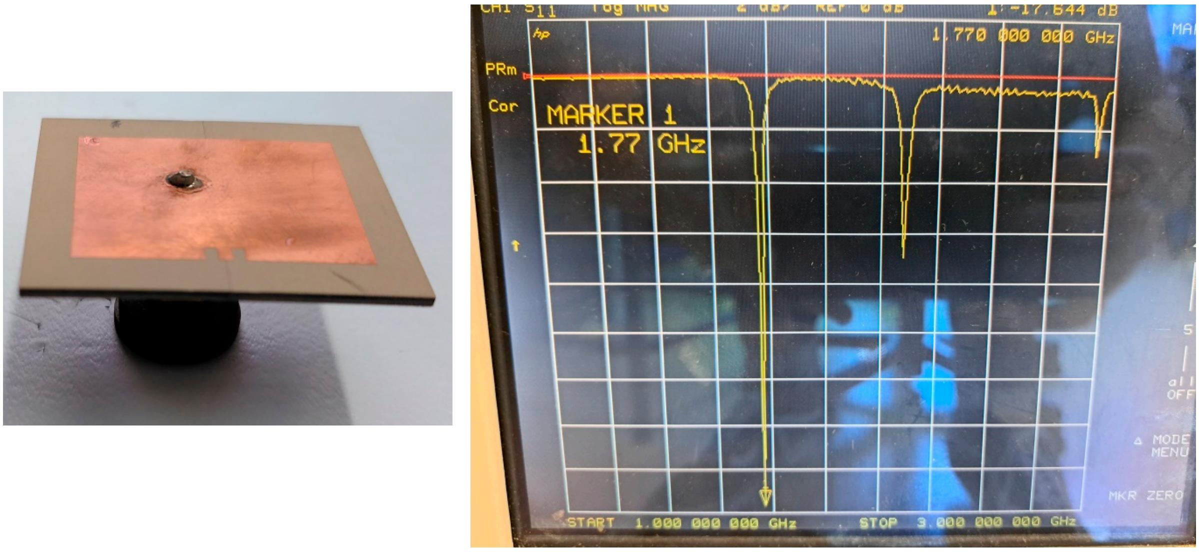

Figure S2 : photo of actual patch antenna (left) and photo of measured reflection coefficient, S11=-17.6dB@1.77GHz (right)

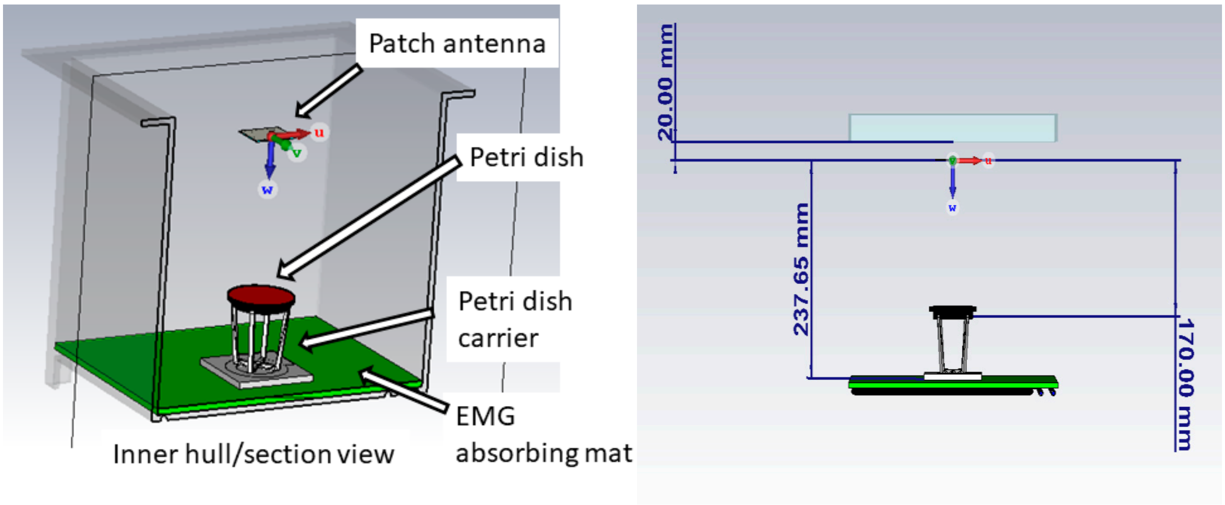

Figure S3 : CST design of RF radiation onto Petri dish (left) and its characteristic dimensions (right)

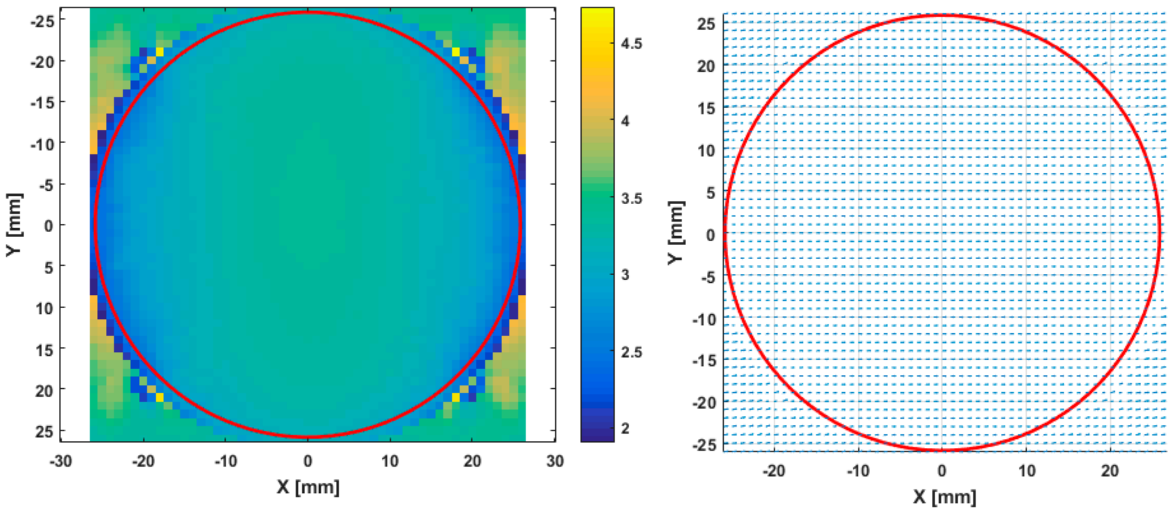

Figure S4 : Electromagnetic power flow [W/m^2] at a distance of 168mm from the antenna (left) and electric field vector in the XY transverse plane (right). The red circle represents the Petri dish (CST simulation, Matlab graph). This simulation gives a qualitative view of power flow homogeneity (left) and electric field polarization (right) on the Petri dish.

Data S1 : Simulation parameters at 1.8GHz

- Patch antenna :
  Approximate dimensions: 42mmx34mm
  Substrate material: RO3006 (Rogers corp.) ceramic-filled PTFE composites
  Simulation values: typical $\varepsilon_r = 6.15$ and $\tan\delta = 0.002$
  Ground plane and patch material: Perfect Electric Conduction (PEC)
- Double-shell box and hood:
  External dimensions: 373mm x 373mm x 337mm (length x width x height)
  Interior dimensions: 289mm x 289mm x 295mm (length x width x height)
  Material: PMMA
  Simulation values: typical $\varepsilon_r = 2.59$ and $\tan\delta = 0.002$
- Thermal insulation :
  Thickness: 30mm
  Material: XPS
  Simulation values: typical $\varepsilon_r = 2.5$ and $\tan\delta = 0.00033$
- Electromagnetic absorber :
  Thickness: 6mm
  Material: RGD-S-124 (Cuming Microwave) filled silicone with high magnetic loss
  Simulation values: $\varepsilon_r' = 21.5$ and $\varepsilon_r'' = 1$ (constructor data)
  Simulation values: $\mu_r' = 5$ and $\mu_r'' = 2.15$ (constructor data)
- Pipe for water ciculation:
  Material: Silicon
  External diameter: 6mm
  Simulation values: typical $\varepsilon_r = 4.2$ and $\tan\delta = 0.$
- Hot water :
  Simulation values: typical $\varepsilon_r = 68.6$ and conductivity S/m $\sigma = 0.33$
- Petri and probe holder :
  Material: PLA
  Simulation values: typical $\varepsilon_r = 2.9$ and $\tan\delta = 0.01$
- Petri dish :
  Material: polystyrene
  Simulation values: typical $\varepsilon_r = 2.4$ and $\tan\delta = 0.$

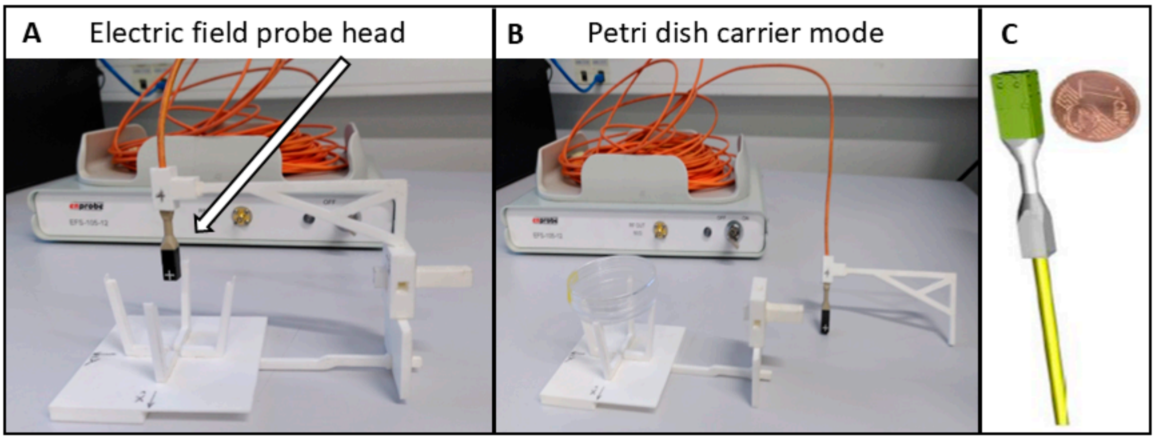

Figure S5: Petri dish holder convertible into electric field probe holder: In the background (A and B) is the converter that transforms the electric field power measured by the probe head into electrical power that can be measured with a spectrum analyzer via the SMA port (gold connector on the front panel). This measuring device is connected by an optical fiber (orange) to the electric field probe, in the foreground, in black with the white + on it. A. The probe head is held by a jib where the Petri dish should be, resting on the four arms visible below the probe. B. The jib is removed and the Petri dish is placed on the arms. Comparison of A. and B. shows the effect of modularity. Figure S3 illustrates the Petri dish support mode. Figure S6 shows the electric field probe support mode. C. is a zoom on the probe head.

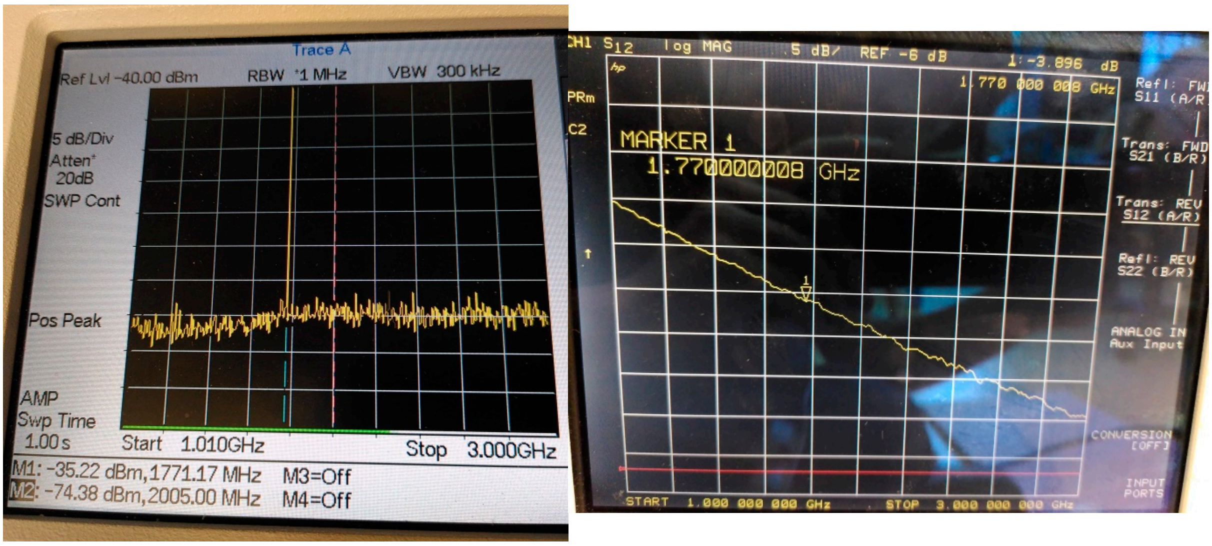

Figure S6: (Left) Power measurement with the spectrum analyzer. The generator output power is +10dBm, the antenna radiates the power inside the radiating box, the electric field probe senses the incoming field amplitude and the spectrum analyzer provides the measured electrical power P_{MES}. This electrical power is then transformed to a power flow value P_{EMG}. (Right) VNA measurement of the S12 transmission coefficient, A_{CABLE}, of the SMA cable linking the generator and the antenna feeding SMA port. S12=-3.896dB@1.77GHz.

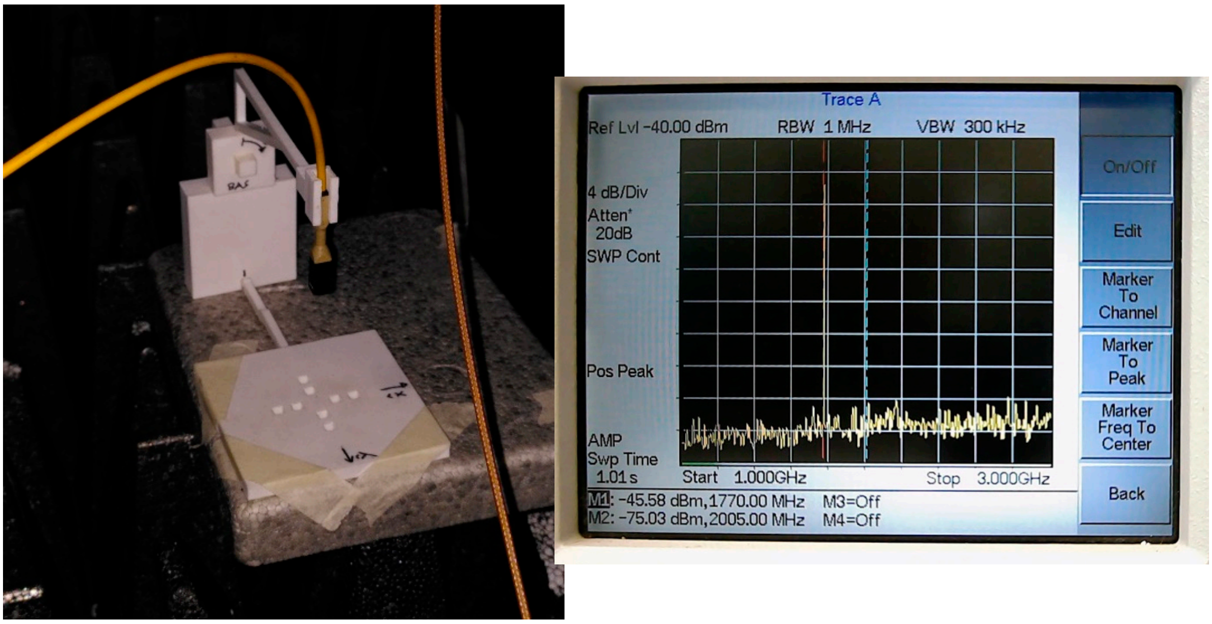

Figure S7 : The indirect field measurement device in the anechoic chamber. The electric field probe is installed on its carrier. The photo (left) shows the device used to make an indirect field measurement: placed in the anechoic chamber, outside and right next to the radiation box during cell illumination, it measures the electromagnetic environment. The spectrum analyzer (right) connected to the probe gives a power peak at 1771.17MHz, which corresponds to the cell illumination signal when it is emitted, and verifies that there is nothing for the control samples. It also gives the power of any unwanted signals between 1 and 3 GHz. In this case, there's nothing above -70dBm. The actual amplitude of the power peak (in this case -45.58dBm for P_{gene}=+10dBm) depends on generator output power but is here irrelevant. This is only an indirect measurement whose function is only to verify the presence or absence of a signal at a frequency between 1 and 3 GHz.

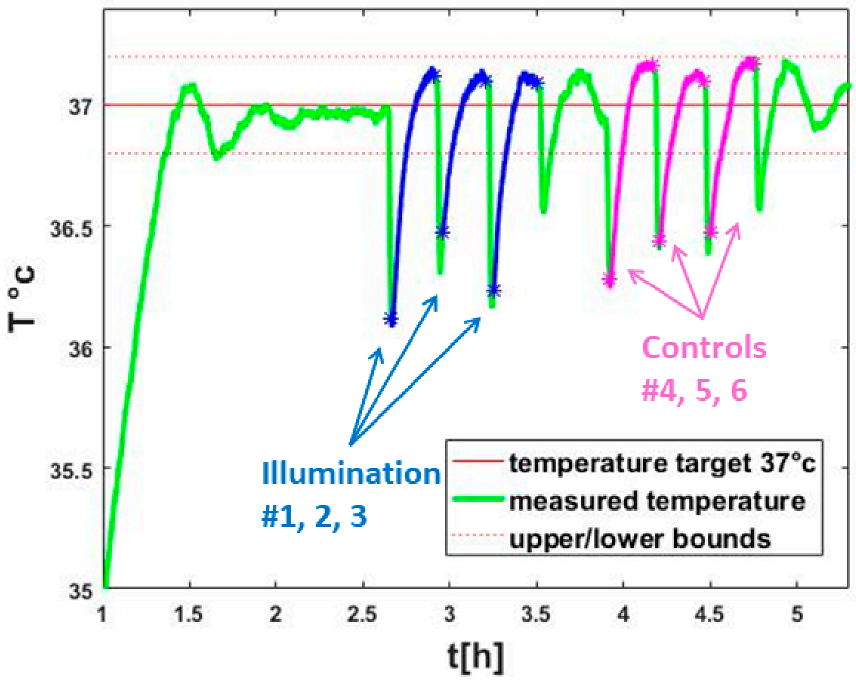

Figure S8: Temperature monitoring during 6 consecutive petri dish RF exposures. The initial step (hours 0 - 2.5) is to arrive at the set temperature of 37°C before beginning the experiment at 2.5 hours. The transient small temperature drops occur each time the exposure box is opened for insertion of subsequent samples. The temperature never goes **above** set point of 37.2 degrees.
